# A Simplified Methodology for Solvent Screening in Selective Extraction of Lipids from Microalgae Based on Hansen Solubility Parameters

**DOI:** 10.3390/molecules30224428

**Published:** 2025-11-16

**Authors:** Costas Tsioptsias, Stefania Mitis, Alexandra Rentzela, Kalitsa Alvanou, Dimitra Kelesi, Christos Manolis, Anastasia Stergiou, Sotirios D. Kalamaras, Petros Samaras

**Affiliations:** 1 Department of Food Science and Technology, Alexandrian University Campus at Sindos, International Hellenic University, 57400 Thessaloniki, Greecenatasastergiou@hotmail.com (A.S.); samaras@ihu.gr (P.S.); 2 Laboratory of Animal Production and Environmental Protection, Faculty of Veterinary Medicine, School of Health Sciences, Aristotle University of Thessaloniki, 54124 Thessaloniki, Greece; skalamaras@vet.auth.gr

**Keywords:** algae, biodiesel, solvent extraction, selective extraction, Hansen Solubility Parameters, lipids, glycerides

## Abstract

Microalgae are considered a potential source of fatty acid esters that are suitable for biodiesel production. However, a principal bottleneck in lipids extraction is related to the selection of appropriate solvents in order to obtain an efficient process. In this work, a simple methodology based on Hansen Solubility Parameters (HSP) was developed, aiming to solvent screening towards selective extraction of lipid compounds: main parameters that were considered for an optimum solvent included the partitioning of free fatty acids and other non-desired solutes, e.g., pigments and phospholipids, as well as the minimum water dissolution. The method takes into account the affinity of a candidate solvent with desired and non-desired solutes along with their relative differences. A large number of solvents (>5000) were scanned by this method for their capacity to selectively extract fatty acid esters from microalgae biomass, and hexane proved to be among the optimum solvents. This prediction was supported by the Snyder’s polarity index as well as ab initio quantum mechanical Density Functional Theory (DFT) calculations of the Gibbs free energy of solvation and partition coefficients. Moreover, model validation carried out by liquid–liquid extraction of algal liquor with hexane and other solvents, and measurement of lipids allocation using paper chromatography and spectroscopy. Low lipids yield was observed, while the extract was enriched in fatty acid esters. A critical discussion is provided regarding the low yield ratios and potential implications due to overestimation of lipids content in microalgae.

## 1. Introduction

Selective extraction has a tremendous range of applications including analytical chemistry [1,2], materials science [3], production of high added value products of organic (extraction from herbs [4,5]) or inorganic origin (metals [6]), recovery of valuable substances (lithium from used batteries) [7], etc. However, the selection of a solvent suitable for extraction of target compounds represents rather a difficult task. A straightforward but cost- and time-consuming approach for the selection of an appropriate solvent, is certainly to test various solvents under laboratory experiments: for example nine different hydrophobic eutectic solvents have been studied for the selective extraction of Molybdenum and Rhenium [6]. On the other hand, solvent screening based on a reliable theoretical approach could save time and costs compared to laboratory practices.

Different approaches have been reported for solvent screening used in various applications. There is increased interest for the Conductor-like Screening model (COSMO), which is a thermodynamic model that is based on quantum mechanics and Density Functional Theory (DFT) calculations. COSMO-based approaches have been extensively applied for solvent screening in numerous applications such as CO_2_ capture [8,9], minimum energy demand in extraction–separation processes [10], separation of aliphatic from aromatic compounds [11], extraction of natural organic compounds [12,13], finding solvents of low toxicity [14], etc. However, COSMO-based methods require the use of specialized software, which is not freely available even for academic use, while it may be time-consuming, depending on the hardware that is available for the calculations. In addition, a rather solid background in physical chemistry is required in order to properly build and perform such calculations. Therefore, the utilization of such software, although reliable, is not readily applicable.

An alternative method for solvent screening is based on HSP [15]. HSP are related to the cohesive energy density and account for dispersion forces, polar interactions, and hydrogen bonding; these parameters are widely used by academics and industrial scientists in order to predict the solubility of various substances such as polymers [16,17], paints [15], pharmaceuticals [18], or for several other applications such as finding solvents with low toxicity [19], extraction of chlorophyll from algae [20], extraction used for analysis [21,22,23], solvents for depolymerization of lignocellulosic biomass [24], green solvents for preparation of semiconductor thin films for optoelectronic devices [25], and other. The concept behind HSP is that “like dissolves like” [18]. Although HSP do not always provide accurate data [18], they exhibit a remarkable prediction capacity of solubility taking into account their simplicity. In addition, HSP values for thousands of substances are already available, the vast majority of which have been calculated based on thermodynamic (experimental) solubility data [15]. Thus, it is not surprising that such a simple approach typically yields reliable predictions. In addition, simple group contribution methods can be used for the estimation of HSP values for less known substances [26]. Apparently, solvent screening techniques based on HSP are much faster and simpler than COSMO-based approaches, do not require specialized software or hardware and can be used by those that have not a strong chemistry background. In addition to COSMO-RS [27], other thermodynamic models can be used such as UNIFAC (Universal Quasichemical Functional-group Activity Coefficients) [28] or NTRL-SAC (Non-Random Two-Liquid Segment Activity Coefficient) [29] to predict fluid phase equilibria for a range of pressures and temperatures. Nevertheless, the computational accuracy of these models is better than that of HSP which provides qualitative information on solubility, while empirical equations are accounting for temperature effects. A short comparison of alternative models is presented in Table S1 of the Supplementary Material.

The research interest for algal biomass is continuously growing due to its numerous potential applications. One such application is the production of biodiesel from lipids contained in microalgae; this stage represents the bottleneck in the entire microalgae valorization line and the selection of an appropriate solvent receives considerable efforts for the elaboration of low-cost and efficient extraction techniques. Solvent screening for lipids extraction from algae has been performed with calculations of the partition coefficient by COSMO-RS (Conductor-like Screening model for Real Solvents) along with other parameters associated with toxicity, environmental, and safety issues [30,31]. Solvent screening based on COSMO has also been applied for extraction of pigments (chlorophylls and carotenoids) from algae [32,33] and for fractionation of wet microalgae [34]. Typically, a good solvent is selected based on its solvating properties of target solutes (e.g., lipids) without accounting for the interactions of the solvent with other potential (non-desired) solutes. For example, it has been reported that 2-butanol is very effective for extracting 99.4% of the lipids from wet algae although it simultaneously extracts 82.6% of unwanted carotenoids [35].

Algorithms recently reported for solvent screening towards lipid extraction based on COSMO-RS [30] or HSP [23] are focused on the prediction of appropriate solvents having a high affinity with the desired solute(s), considering in addition factors related to safety, toxicity, etc. In these methods, solvent pre-screening is carried out based on the thermodynamic solubility data, and then the candidate solvents are further examined based on their “green” properties. As a result, various environmentally friendly alternative solvents are examined, such as supercritical fluids, ionic liquids (ILs), and deep eutectic solvents (DES) including natural deep eutectic solvents (NADES) in addition to more traditional organic solvents. These solvents are studied due to their unique properties such as recycling, low toxicity, lack of flammability, etc. Approaches for the selection of green solvents are useful since solubility and environmental issues are taken into account. However, the main drawback of these techniques is the presence of a great number of solutes in the extract not accounted for in the models, i.e., a solvent with strong affinity for a desired solute, may have the same or even greater affinity with other non-desired solutes. Therefore, the selection of an optimum solvent by these methods can provide a chemical resulting in an extract enriched with both desired and non-desired compounds.

In other words, the strong solvating property of a solvent for a target solute is the primary condition for being considered a suitable candidate in extraction. However, it is not the only condition, since an appropriate solvent should not exhibit strong solvating properties for other potential co-extractable compounds. Such an aspect is rarely taken into account during solvent screening. The extracted lipids fraction from microalgae usually contains a range of compounds which represent the desired raw materials for biodiesel production such as free fatty acids or triacylglycerides (TAGs). However, other unwanted lipids may be found in the extract, such as phospholipids, which due to their strong polar phosphate group act as emulsifiers creating problems in the biodiesel overall preparation process that have to be removed in a prior treatment stage. In addition, the presence of phospholipids and chlorophyll in produced biodiesel, results in the formation of inorganic ash in the combustion engine, therefore burdening the exhaust gases quality and the performance of the engine, while impacting the fuel’s specifications.

Therefore, the production of biodiesel in an acceptable quality requires additional purification steps after the extraction of lipids from microalgae. The selective extraction of fatty acid esters from microalgae could assist in reducing cost and simplifying the overall biodiesel production process by reducing post-processing requirements of raw extract. Under these conditions, a simplified approach for solvent screening focusing on selective extraction of target lipids from microalgae would greatly enhance process performance. The objective of this work is to develop a simplified methodology, based on HSP, for solvent screening in selective extraction processes and the examination of its application for the selective extraction of fatty acid esters from microalgae. Furthermore, the work aims to assess the validation of the proposed methodology through thermodynamic calculations based on DFT and its laboratory experimental certification for lipids extraction from microalgae biomass samples. 

## 2. Results and Discussion

The selection of the four solvents (hexane, ethyl acetate, DMCHA, and 1-pentanol) took place for this study according to the following: For the extraction of lipids, solvents of medium to low polarity are required. As shown in Section 2.1, the particular solvents cover a broad range of (suitable medium to low) polarity and thus allow for a comprehensive examination of the extraction of lipids from microalgae. In addition, hexane and ethyl acetate are common organic solvents used for oil extraction, and although traditionally are of fossil fuels origin, they can be produced by biobased techniques. Furthermore, they are cheap solvents and not particularly toxic. DMCHA exhibits the interesting property of polarity switching and recently has attracted attention for extraction and separation of lipids from microalgae [36,37]. Fatty acid esters exhibit a small polar part (due to ester groups) and a long non-polar part. Similar structure is exhibited by fatty alcohols, e.g., dodecanol, thus, such alcohols are expected to exhibit affinity and good solubility properties for lipids. However, the separation of these alcohols from fatty acid esters is difficult to accomplish due to their high boiling points. In addition, 2-butanol, which has a lower non-polar part, has been proposed as candidate solvent for extraction of lipids from microalgae [35]. Thus, 1-pentanol seems to be a solvent with compromising properties between separation ability and affinity with lipids. Besides these four solvents, also, the solvent (mixture of chloroform, methanol, and water) that is used in the Bligh–Dyer method for the determination of total lipids was tested experimentally.

From the five solvents that were tested experimentally, two of them, namely hexane and 1-pentanol (and water) were also tested theoretically by DFT. These solvents represent the optimum and worst case substances according to the algorithm. DMCHA is not included in the list of solvents in the software we used and the corresponding solvation model, thus it was not possible to perform the DFT calculations for this solvent.

Specific solutes were selected in this study for the DFT calculations as common representatives of microalgae lipids, including glycerides, sterols, pigments, and phospholipids, based on algae cell composition, which, however, depends on cultivation conditions, e.g., in the same study, the same *Chlorella sorokiniana* strains cultivated in a 20 L laboratory tubular photobioreactor and in a large-scale 25,000 L reactor exhibited different carotenoids and fatty acids content/profile [38]. In the former study, ergosterol was found to be the dominant sterol, however, β-sitosterol is also present in algae [39]. In addition, these sterols have similar structures (and their HSP values are very close). In another study, it was reported that the content of chlorophyll a is higher than that of chlorophyll b, and lutein seems to be the major carotenoid in a *Chlorella* culture, in addition to other compounds such as alpha and beta carotene [38]. Moreover, *Chlorella sorokiniana* contains numerous phospholipids including the one considered for the DFT calculations, i.e., phosphatidylethanolamine [40]. Also, oleic, palmitic, linoleic acid, and their esters are common in *sorokiniana* [38]. Since DFT is time-consuming, one representative substance for each group (glycerides, phospholipids, etc.) was examined.

It should be underlined that the suggested algorithm can have a wide range of applications, considering the solvents affinity with desired and non-desired solutes. In the certain work, this approach is applied for the investigation of a solvent capacity to the extraction of lipids from microalgae, due to their importance for biofuels production; nevertheless, the same technique can be applied for the estimation of lipids extraction from a wide range of species beyond *Chlorella sorokiniana*, since microalgae species usually present similar or even the same lipidic and other fractions, at relevant concentrations.

### 2.1. Application of the Proposed Methodology to the Selective Extraction of Fatty Acid Esters from Microalgae

The individual parameters for the estimation of HSP of certain desired and non-desired representative solutes are shown in Table 1; these parameters were retrieved from the relevant literature [41].

Four fatty acid esters (glycerides) along with five representative non-desired solutes were selected to be studied in this work, covering a broad range of groups, i.e., sterols, pigments, and phospholipids. For each group, solutes with different HSP were chosen: β-sitosterol and cholesterol exhibit similar HSP thus only one was chosen for the groups of sterols. Similarly, phosphatidylcholine has similar HSP with phosphatidylethanolamine, thus for the groups of phospholipids, only the latter was considered along with phosphatidylserine which has quite different HSP.

Based on the simplified methodology, the overall scores of the five experimentally tested solvents along with their Snyder’s polarity index [42] and the dielectric constant of five candidate solvents, that were experimentally tested, are presented in Table 2. All values of dielectric constant were obtained from the Handbook of Chemistry and Physics [43] except the one of DMCHA that was retrieved from Material Data Safety Sheet [44]. In addition, a file with the scores of 5632 substances is provided as Supplementary Data (Table S2) and the corresponding position of the certain solvents used in this work is included in Table 2. It was observed that in the relevant list, extremely few silanes and mostly fluorinated compounds are ranked within the first 100 positions. Since organic fluorinated compounds are not environmentally friendly this group of compounds was not considered for further analysis. A few amines and a large number of branched alkanes and alkenes were placed in the next positions, following fluorinated compounds, containing typically hexanes/hexenes, heptanes/heptenes, and octanes/octenes. Specifically, hexane is classified in the position 188 out of 5632 substances which is a rather good ranking in the overall list. DMCHA is ranked at a lower position but higher than ethyl acetate. 1-pentanol is predicted to be the worst solvent among the four candidate solvents for selective extraction of lipids from algae.

**Figure 1:**
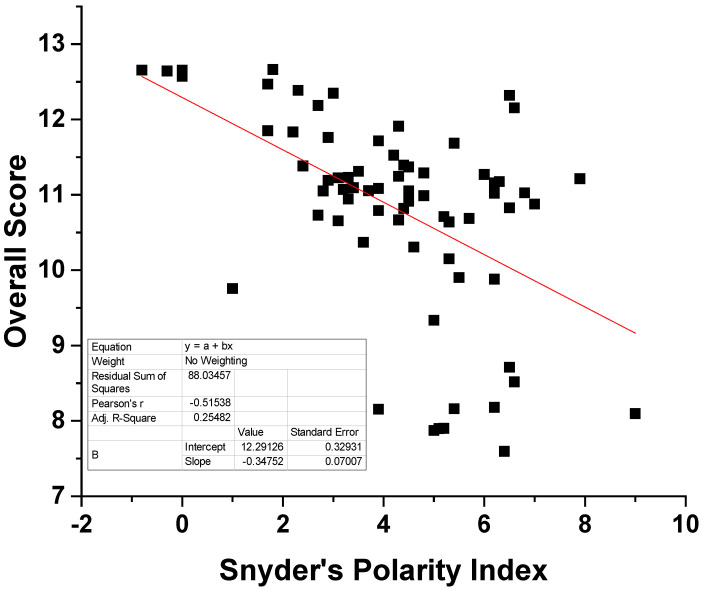
Plot of the Snyder’s polarity index against the overall score for 70 solvents.

As can be seen in Table 2, the overall scores seem to be negatively correlated to the dielectric constant. The same trend is observed for the polarity index, although the exact corresponding values for DMCHA and 1-pentanol could not be found. Nevertheless, it seems that the more polar solvents have received a lower score. In order to further validate this hypothesis, the Snyder’s polarity index was plotted as a function of the overall score for 70 solvents of various nature, e.g., hydrocarbons, alcohols, ethers, esters, etc., in Figure 1. In the original paper by Snyder [42], the polarity index of 75 solvents is given, while 70 of them were included in our calculations. The names of the solvents and the corresponding values are presented in Table S3 of the Supplementary Material. As presented in Figure 1, negative correlation trends are obvious and solvents of low polarity generally received higher scores than polar ones. Nevertheless, a linear correlation would not be expected since the overall score is not influenced solely by the polarity of the solvent. In addition, polar solvents most likely would exhibit poor selectivity towards low polarity substances like TAGs. Based on the above, it could be concluded that the proposed algorithm and the corresponding methodology yield scores and solvent rankings that are physically meaningful. The predictions and the reliability of the proposed methodology are further verified in the following, using theoretical and experimental approaches.

### 2.2. Gibbs Free Energy of Solvation and Partition Coefficients

The Gibbs free energy of solvation of five representative solutes, i.e., glyceryl triolate, β-sitosterol, chlorophyll a, phosphatidylethanolamine, and lutein, by water, hexane, and 1-pentanol are presented in Table 3.

As can be deduced from Table 3, the solvation of the fatty acid ester, glyceryl triolate, by hexane exhibits the most negative value. This suggests that hexane has the highest affinity for glyceryl triolate over the other solutes and thus it is likely that hexane is expected to present high selectivity for this compound. On the contrary, 1-pentanol has the highest affinity for chlorophyll as depicted by the lowest ΔG value. In addition, although 1-pentanol presents a high negative value of ΔG for glyceryl triolate, higher absolute values than those of hexane were estimated for the other solutes. This suggests that 1-pentanol exhibits high affinity for all solutes and thus it less likely to present selectivity for fatty acid esters.

Glyceryl triolate has low affinity with water compared to other solutes as depicted by the moderate negative value of ΔG of solvation by water. This is an important parameter that has to be considered in cases of extraction from wet microalgae as wet paste received after, for example, centrifugation with certain water content or in liquid phase, was usually obtained by simple sedimentation processes. Therefore, for the assessment of a solvent capacity to be used in liquid–liquid extraction processes, the corresponding organic solvent/water partition coefficients of the solutes should be determined. These parameters are presented in Table 4 and these values should be interpreted in combination with ΔG of solvation.

The partition coefficients of glyceryl triolate is the highest among the solutes, for both hexane and 1-pentanol, due to the less negative value of ΔG of solvation of this solvent by water and the very negative values for the organic solvents. However, although 1-pentanol exhibits the highest affinity for chlorophyll a, this is not depicted in the corresponding partition coefficient since water exhibits simultaneously strong solvation potential for chlorophyll. However, the hexane/water partition coefficient of glyceryl triolate is much higher than the other solutes, while less severe differences are observed for 1-pentanol/water partition coefficient. As a result, based on the Gibbs free energy of solvation and partition coefficient analysis, it can be concluded that hexane is more likely to exhibit selectivity towards fatty acid esters than 1-pentanol, supporting the corresponding HSP based predictions.

Further insights can be provided by the difference in solvation energies, ΔΔG_solvation_, between the desired and non-desired solutes calculated as ΔΔG_solvation_ = ΔG_solvation, desired solute_ − ΔG_solvation non-desired solute_. These values are presented in Table 5. As can be seen, the solvation energy difference in hexane corresponds to negative values, much lower than the other two solvents, suggesting high affinity for the desired solute and low affinity for non-desired solutes.

### 2.3. Paper Chromatography of Solvent Extracted Microalgae Lipids

**Figure 2:**
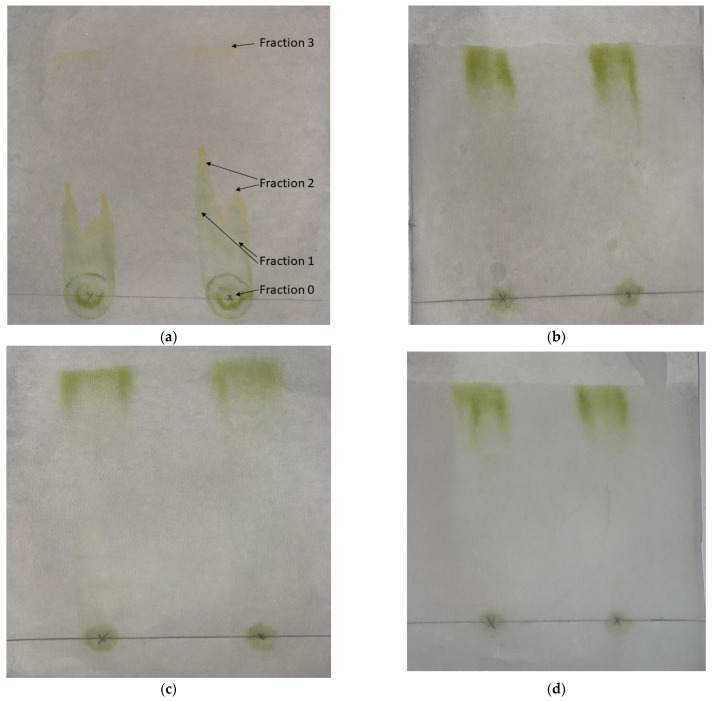
Paper chromatographs of the DMCHA-algae extract obtained with various solvents: (**a**) hexane, (**b**) DMCHA, (**c**) ethyl acetate, and (**d**) 1-pentanol.

Paper chromatography was used for the evaluation of the affinity of various solvents with the solutes that were extracted with DMCHA. Characteristic photos are presented in Figure 2a–d, showing the paper chromatographs of DMCHA-algae extract obtained by four different solvents. From the chromatographs shown in Figure 2b–d it can be deduced that all compounds that were present in the extract were eluted together by the three solvents DMCHA, ethyl acetate, and 1-pentanol. However, as can be seen in Figure 2a, three distinct fractions can be observed in the chromatograph of hexane as the mobile phase, along with the residue (fraction 0). Since the same extract was used in all four cases as the initial sample and therefore the same solutes were casted at the initial point of the chromatograph, and since the static phase is the same (cellulose paper) for all solvents, any observed differences in elution can be attributed to the different interactions of the solutes with the solvent, i.e., the mobile phase. It is therefore apparent that the three solvents, DMCHA, ethyl acetate, and 1-pentanol, exhibit the same high affinity for all solutes in the extract, while hexane presents considerably different affinity for the different solutes, resulting in the formation of different fractions eluted at different time and shown at a different distance from the residual fraction.

**Figure 3:**
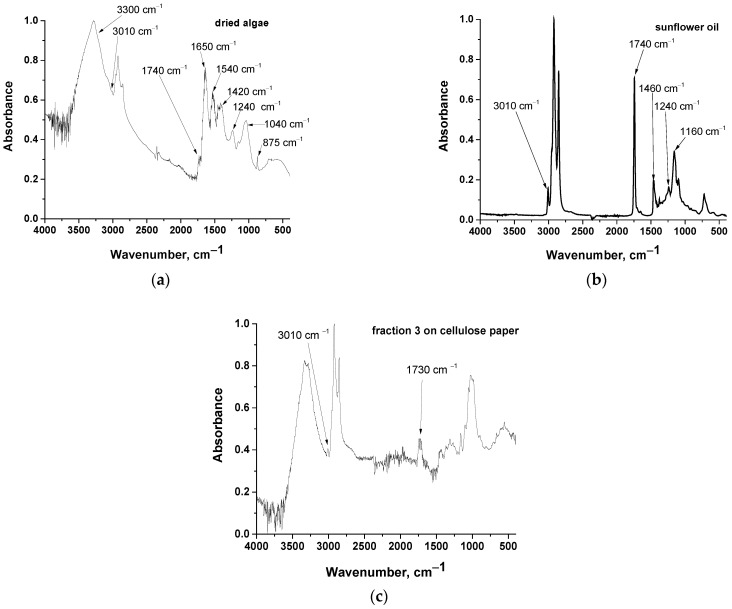
ATR-FTIR spectra of: (**a**) dried algae, (**b**) commercial sunflower oil, and (**c**) fraction 3 (see Figure 2a) on cellulose paper.

Fractions 1, 2, and 3 were further examined by ATR-FTIR; however, a spectrum of acceptable quality was obtained only for fraction 3 (in the other cases the observed signal corresponded to that of cellulose, i.e., the background paper–we also tried to measure it by using paper as background, but again the spectra were not evaluable). The spectrum of fraction 3 on cellulose paper along with the spectrum of a commercial sunflower oil used for simulating algal oil [45,46] and dried algae are presented in Figure 3.

Dried algae present typical absorption bands of cellulose, proteins, and lipids. Specifically, the band at 3300 cm^−1^ can be attributed to O-H stretching in cellulose and N-H stretching in proteins [47]. The band around 2900 cm^−1^ is common for all organic substances and is assigned to C-H stretching [47]. A small peak above 3000 cm^−1^ (specifically at 3010 cm^−1^) suggests the existence of =C-H [47]. Such groups are contained in unsaturated fatty acid esters (lipids). The small peak at 1740 cm^−1^ is characteristic of ester C=O stretching [47] and is attributed to lipids. The bands at around 1650 and 1540 cm^−1^, are, respectively, of the amide I (C=O stretching and primary amide NH_2_ bending) and amide II (C-N stretching and secondary amide N-H bending) bands in proteins [47]. The band at 1420 cm^−1^ is related to various C-H and methylene vibrations [47]. The band at around 1240 cm^−1^ is assigned to P=O stretching [47] and attributed to phospholipids. The band at around at 1040 cm^−1^ is assigned to C-O stretching (present in cellulose and lipids) [47,48]. Finally, the band around 895 cm^−1^ is related to C-H deformation in cellulose [48,49]. Chlorophyll bands overlap with some of the above-mentioned bands.

Commercial sunflower oil exhibits bands at around 3010, 2900, 1740, 1400, 1240, and 1160 cm^−1^ which are assigned, as above, respectively, to =C-H stretching, C-H stretching, C=O stretching, various C-H vibrations, P=O stretching, and C-O stretching. Thus, in the commercial sunflower oil, phospholipids are present along with fatty acid esters. In the spectrum of fraction 3 on the cellulose paper (Figure 3c) major peaks correspond to bands of cellulose. As mentioned above, bands corresponding to C-H and C-O stretching overlap and are common in both cellulose and lipids. Nevertheless, certain peaks that correspond to C=O stretching at 1730 cm^−1^ and =C-H stretching at 3010 cm^−1^ are clearly detectable and are attributed to glycerides rather than to cellulose. The above findings confirm that fraction 3, i.e., the fraction that was not separated from the solvent and followed the solvent up to the upper point of the paper chromatograph, consists mainly of fatty acid esters, indicating the high capacity of hexane for extraction of these compounds from microalgae.

As a first conclusion, the prediction of the proposed simplified methodology based on HSP, is experimentally confirmed by paper chromatographs and by FTIR spectroscopy results.

### 2.4. Extraction from Algae with Hexane and Chloroform/Methanol Mixture

**Figure 4:**
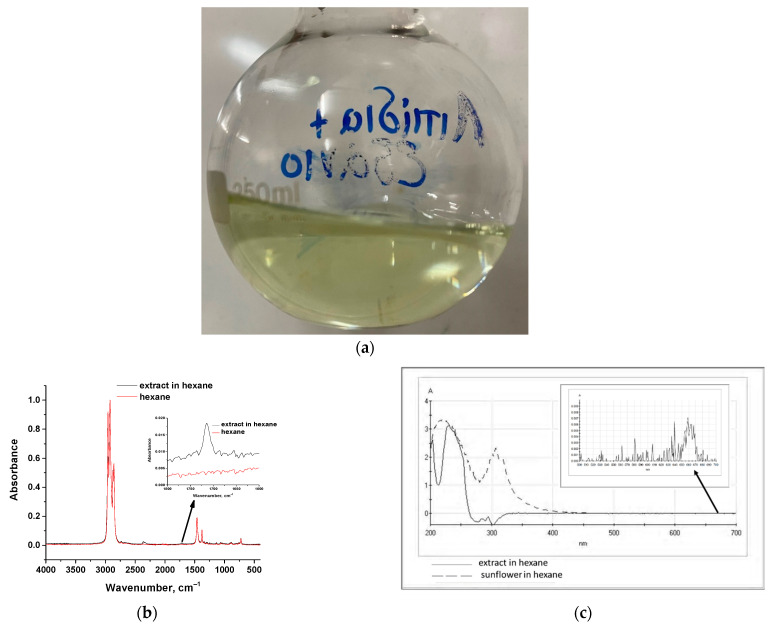
(**a**) Photo of the hexane-extract of microalgae liquor, (**b**) ATR-FTIR spectra of pure hexane and the hexane-extract of microalgae liquor, and (**c**) UV–Vis spectra of the hexane-extract of microalgae liquor and of sunflower 50% *v*/*v* in hexane.

Hexane was used as a suitable solvent for the selective extraction of fatty acid esters from a 2 g/L microalgae liquor; liquid–liquid solvent extraction was carried out for 4 h and a photo of the extract is presented in Figure 4a.

A yellowish color is observed in the final liquor, without strong green shades, compared to the corresponding extract obtained with DMCHA that had an intense green color received even by the first 30 min of extraction. The corresponding ATR-FTIR spectrum of the hexane-extract along with the spectrum of pure hexane are presented in Figure 3b. The absorption peaks of lipids in the spectrum are very weak due to the dilute solution. In addition, C-H peaks overlap with the respective ones of hexane. However, C=O stretching at around 1715 cm^−1^ is identified clearly (embedded diagram in Figure 4b). Moreover, UV–Vis spectra of the hexane-extract and a 50% *v*/*v* solution of sunflower oil in hexane are presented in Figure 4c. It can be deduced from this figure, that the two solutions exhibit strong absorption in the 200–250 nm region. Moreover, in the embedded diagram in Figure 4c, it can be observed that the hexane-extract exhibits a very weak absorption at 660 nm, attributed to the presence of a minor amount of chlorophyll.

The yield of extraction by hexane was estimated to be equal to 1.5 ± 0.3 g of fatty acid esters per 100 g of dry algae while the corresponding recovery of hexane after distillation at atmospheric pressure was 76 ± 6%. The algae sample exhibited a total lipids content of 12.5% as measured by the Bligh–Dyer method. Thus, the extraction efficiency was 12% (1.5/12.5 × 100). The extract yield and efficiency can be enhanced by cultivation of microalgae under appropriate conditions, including selection of appropriate strains, cultivation under N-deficiency diet, and utilization of consecutive extraction stages using low solvent volumes and increased temperature and time of extraction. Nevertheless, low yield of extraction achieved in this work, can be attributed to various reasons, such as the following: (a) selective extraction of fatty acid esters; (b) extraction by hexane performed in wet samples; however, it has been reported [50] that the determination of lipids using two different methods provides lower values in wet samples than in dried samples due to lower extraction efficiency in wet samples; (c) reported values for lipids content in dried algae are commonly based on methods such as the Bligh–Dyer method for the determination of total lipids, i.e., sterols, phospholipids, and most likely chlorophyll, besides fatty acid esters. As a result, simultaneous co-extraction of other compounds is taking place, resulting in an over estimation of the lipids content of algae.

**Figure 5:**
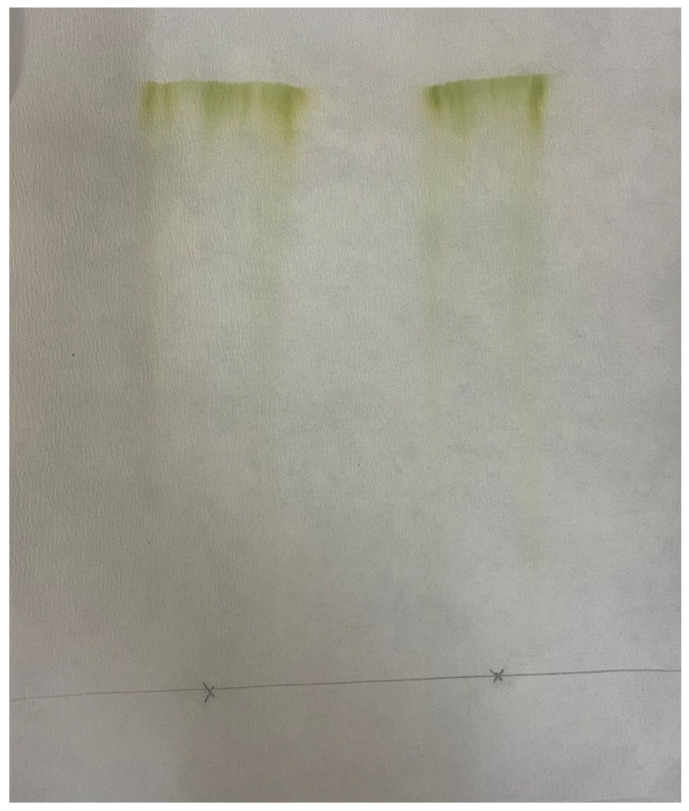
Paper chromatograph of the DMCHA-extract by using a methanol/chloroform/water mixture in a volume ratio of 2/1/0.8 as solvent for the elution.

As mentioned above, a method commonly used for total lipids determination is the Bligh and Dyer method utilizing a methanol/chloroform/water mixture in volume ratios of 2/1/0.8 [51]. In order to compare hexane extraction capacity to the standard solvents mixture, analysis by paper chromatography of the DMCHA-extract using this solvent mixture as mobile phase, was performed. The paper chromatograph received is presented in Figure 5.

**Figure 6:**
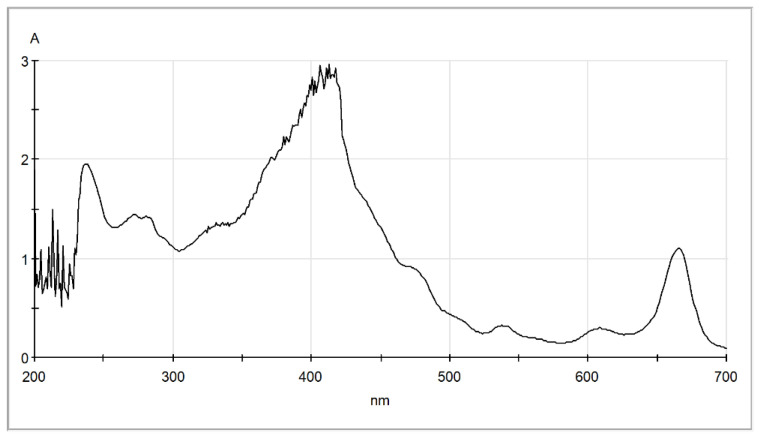
UV–Vis spectra of the extract obtained by the Bligh–Dyer chloroform/methanol/water one-phase mixture.

As can be seen, this solvents mixture exhibits high affinity for all solutes including chlorophyll, and therefore it can be assumed that results in an overestimation of the total lipids in algae. The corresponding scores estimated by the suggested simplified method for this mixture are included in Table 2. Based on these data, the solvents mixture was rejected during screening, since its SCORE 1 value is zero, and is not suitable for selective extraction of fatty acid esters from algae. In order to further support the argument associated with potential overestimation of lipids content, the extract of wet algae received by the methanol/chloroform/water solvent mixture was examined by UV–Vis spectrophotometer and the corresponding spectrum is presented in Figure 6. As shown, the extract has an absorption peak around 240 nm, similar to the corresponding hexane extract and sunflower oil. Absorption in this region is related to hydroxy, carbonyl, and ester groups and possibly to linoleic acid [52]. The sunflower oil, as shown in Figure 3c, exhibited high absorbance around 310 nm, which is typical for polyphenols found in vegetable oils [52]. However, the extract from the chloroform/methanol/water one-phase mixture presented a high absorption peak at around 415 nm, typical for carotenoids [52] such as lutein [53]. Moreover, other smaller peaks are observed at around 610 nm and 660 nm typical for chlorophyll a and b [53]. These observations support the assumption that the Bligh–Dyer extract contains yellow (carotenoid) and green (chlorophylls) pigments, in addition to lipids that are expected to contribute to an overestimation of the total lipids content.

## 3. Theoretical Calculations

### 3.1. Methodology for Solvent Screening for Selective Extraction Based on HSP

The background and the development of the proposed methodology is presented in a general form for solvent screening towards selective extraction applications, followed by the implementation of the method in the specific application for selective extraction of fatty acid esters from microalgae. Briefly, the various candidate solvents are classified regarding their selectivity for a desired solute(s) based on a total score which is calculated by three sub-scores. Nevertheless, the affinity of a solvent to both desired and non-desired solutes is accounted for, in order to identify the solvent that results in an extract that is enriched in the target compounds.

According to HSP theory, every solvent can be assigned its HSP. If the HSP values between two substances are similar then they are compatible, if they are dissimilar then they are non-compatible. This encapsulates the intuition that “like attracts like”. Based on that, it can easily be calculated how alike two molecules, e.g., 1 and 2, are from their HSP distance R_a_ defined as the following:

(1) $$R_{a}=\sqrt{4{\left(\delta_{d,solute}-\delta_{d,solvent}\right)}^{2}+{\left(\delta_{p,solute}-\delta_{p,solvent}\right)}^{2}+{\left(\delta_{hb,solute}-\delta_{hb,solvent}\right)}^{2}}$$

where

$\delta_{d}$: the dispersion HSP of the solute or solvent in MPa^1/2^;
$\delta_{p}$: the polar HSP of the solute or solvent in MPa^1/2^;
$\delta_{hb}$: the hydrogen bonding HSP of the solute or solvent in MPa^1/2^.

Small values of $R_{a}$ suggest that the two substances have similar Hansen parameters, and they are likely to be miscible or compatible while large values correspond to two substances having different Hansen parameters and are unlikely to be miscible or compatible. Therefore, for a suitable solvent that can be used for selective extraction of a target compound, the lower the $R_{a}$ value, the better the solvent is. Nevertheless, HSP values of solvent, target solutes, and other potential extractable non-desired solutes must be known. Certain methods have been reported for the estimation of HSP of particular compounds that are not found in the literature [26].

The calculation of the first sub-score is carried out considering the relative distance of each non-desired solute relative to the desired solutes calculated as:

(2) $${relative\;distance}_{i}=\frac{R_{a,i}}{Average\;R_{a,desired\;solute}}$$

where

$R_{a,i}$: the value of $R_{a}$ of the ith non-desired solute;
$Average\;R_{a,desired\;solute}$: the average value of $R_{a}$ of all desired solutes.

A high value of ${relative\;distance}_{i}$ suggests that the solvent is likely to be a good solvent for the desired solute and a poor solvent for the ith non-desired solute. Nevertheless, prior to the estimation of the average $R_{a,desired\;solute}$ specific consideration should be taken for the values of each individual solvent $R_{a}$. Large values correspond to solutes most likely immiscible, and therefore these compounds should be excluded from the calculations. A threshold for including a pair of solvent-desired solutes in the calculation is 10, which is considered typical to express potential solubility, i.e., at an $R_{a}$ value lower than 10 MPa^1/2^ the desired solute will be soluble in the solvent. If the $R_{a}$ value is higher than 10 MPa^1/2^ then this particular solvent should be rejected since it is not expected to dissolve the desired solutes.

SCORE 1 of the solvent is then calculated as the square root of the sum of the relative distances of all solutes:

(3) $$SCORE\;1=\sqrt{\sum_{1}^{i}{relative\;distance}_{i}}$$

SCORE 1 is useful for a rough estimation of solvent–solutes affinity, but it requires further improvement in certain conditions, according to the following: If the value of $R_{a}$ for the desired solute and solvent is 8 MPa^1/2^ and for a non-desired solute is 16 MPa^1/2^, this means that the relative distance is 2, which can be considered low. However, this solvent can dissolve the desired solute but most likely will not dissolve the non-desired solute as is suggested by the $R_{a}$ values. On the contrary, in a case in which the value of $R_{a}$ for the desired solute and solvent is 2 MPa^1/2^ and for a non-desired solute is 6 MPa^1/2^, this means that the relative distance is 3, which is higher than previously. This can lead to a misleading result that this solvent is more selective than the previous one. However, this is not the case, since in the latter case, the $R_{a}$ values are both lower than 10 MPa^1/2^ thus, the solvent is likely to dissolve both the desired and non-desired solute. Thus, the SCORE 1 will be corrected by adding the SCORE 2. The second sub-score is suggested to account for these discrepancies and is calculated according to the following procedure: when the value of $R_{a,i}$ between the ith non-desired solute and the solvent is lower than or equal to 10 MPa^1/2^, it receives 0, while at higher values its scoring is 1. SCORE 2 is estimated then by the following equation:

(4) $$SCORE\;2=\;\sum_{1}^{i}f(R_{a,i})$$

where

(5) $$\begin{array}{cc} f\left(R_{a,i}\right)=0 & if\;R_{a,i}\leq 10\\ f\left(R_{a,i}\right)=1 & if\;R_{a,i}>10 \end{array}$$

Optionally, a third sub-score (SCORE 3) can be calculated when the suitable solvent should have low affinity with water. The third sub-score is estimated as the square root of the $R_{a}$ distance between water and the candidate solvent ($R_{a,\;solvent/water}$):

(6) $$SCORE\;3=\;\sqrt{R_{a,\;solvent/water}}$$

The square root in SCORE 1 and SCORE 3 is used so that all three scores are of the same order of magnitude and exhibit typical values in the range 1–10.

The overall score is the sum of the three sub-scores:

(7) overall SCORE=SCORE 1+SCORE 2+SCORE 3

The higher the score of a solvent, the higher the probability that the solvent will exhibit selectivity for the desired solutes over the non-desired ones.

The implementation of this methodology in the screening of more than 5000 substances was carried out, utilizing HSP of substances retrieved from [15,41]. Substances with melting point > 5 °C or boiling point < 40 °C were excluded from the analysis, in order to study substances that are liquid at room temperatures. The corresponding pre-screening resulted in 5632 candidate liquid solvents. It should be underlined that isomers of the same compound may exhibit different HSP values, depending on the type of isomerism, and therefore the affinity of a certain solvent can be identified even between different isomers by the proposed method.

### 3.2. Ab Initio DFT Calculations of Gibbs Free Energy of Solvation and Partition Coefficients

In order to check some of the predictions based on HSP and in order to have an estimation of the partition coefficients of representative solutes in some solvents of interest, DFT calculations were performed. Specifically, DFT calculations were used for the estimation of the Gibbs free energy ΔG of solvation of target solutes (desired and non-desired) found in microalgae, by hexane, 1-pentanol, and water. In addition, Gibbs free energy of solvation can be used for the estimation of the organic solvent/water partition coefficient for these solutes. Target solutes that were considered include glyceryl triolate (fatty acid ester) as a representative desired solute, chlorophyll a (pigment), β-sitosterol (sterol), lutein (pigment), and phosphatidylethanolamine (phospholipid) as representative non-desired solutes. Justification for the selection of the solvents and solutes is provided in Section 2.

The structure of the various solutes was drawn or inserted as SMILES (Simplified Molecular Input Line Entry System) in the freely available Avogadro software (Windows version 1.1.0) [54,55]. SMILES is a special chemical notation of molecular structures which allows easy conversion to 3D structure. The same software was used to generate the initial input file with the non-optimized atom XYZ coordinates of each solute. All calculations were performed using the freely available ORCA software (version 6.0.1) [56,57,58]. This ORCA version uses the libint2 library [59] for the computation of the 2-el integrals and has been built by the support of libXC version 6.2.2 [60]. The BP86 [61,62] functional was used for the calculations since it has been reported to be accurate for frequency and geometry optimization calculations [63]. Similarly, the def2-TZVP(-f) [64] basis set was used along with the def2/J [65] auxiliary basis. This functional and basis set are considered reliable for geometry and frequency computations [63] even without applying a dispersion correction. Other common functionals could be used, e.g., B3LYP, along with typical dispersion corrections such as D3 or D4. A more detailed analysis of ORCA calculations can be found in [66,67,68,69,70,71]. The above-mentioned non-optimized atom coordinates were used as the input file for simulating the geometry of the molecules in vacuum. Then, the optimized coordinates in vacuum were used as the input for the determination of optimum optimization of each solute dissolved in a solvent (i.e., hexane, water, and 1-pentanol). For the solvation, the Universal Solvation Model (SMD) [72] was used in ORCA which is an improvement of the Conductor-like Polarizable Continuum Model (CPCM) [73].

The main result of interest from the performed geometry optimizations, either in vacuum or in a certain solvent, is the single point energy value (${SPE}_{vacuum}$ and ${SPE}_{solvent}$, respectively). The Gibbs free energy of solvation of each solute in each solvent is calculated through the following equation:

(8) $${\Delta G}_{solvation}={SPE}_{solvent}-{SPE}_{vacuum}$$

Once the Gibbs free energies of solvation are known, then, the organic solvent/water partition coefficient of each solute (${logP}_{organic\;solvent/water}$) at a temperature T, in resemblance to the 1-octanol/water partition coefficient, can be calculated as follows [74]:

(9) $${logP}_{organic\;solvent/water}=\frac{-({\Delta G}_{solvation,organic\;solvent}-{\Delta G}_{solvation,water})}{2.303RT}$$

The Gibbs free energies of solvation were calculated at a temperature of 298.15 K and pressure of 1 atm.

## 4. Experimental

### 4.1. Materials and Instruments

Microalgae species (*Chlorella sorokiniana*) were cultured in a tubular photobioreactor with anaerobic digestate as substrate following the operation mode as described in [75]. A Nicolet 380 Attenuated Total Reflectance Fourier Transform Infrared (ATR-FTIR) spectrometer along with a HELIOS Gamma UV–Vis spectrophotometer (Thermo Electron Corporation, Waltham, MA, USA) were utilized for the analysis of dry cells and hexane extracts. Microalgae cell disruption was carried out through a Bandelin Sonoplus ultrasound generator (Berlin, Germany). Extraction solvents included n-hexane (Carlo Erba, ACS grade, Milano, Italy), ethyl acetate (Panreac, 99.9%, Barcelona, Spain), n,n dimethylcyclohexyl amine (DMCHA, Roth, >99%, Karlsruhe, Germany), and 1-pentanol (Fluka, >98%, Buchs, Switzerland), while a commercial edible sunflower oil was also used as the reference sample.

### 4.2. Paper Chromatography

Initially, 80 mL of algae liquor with a solids content of 1.5 ± 0.5 g/L were ultrasonicated with a power of 62.5 W/L for 40 min (corresponding to 1.5 kJ/mL) to ensure disruption of cell walls making their content accessible by the solvents for the following extraction of the various lipid compounds. Verification of cell disruption was based on the color of the extract in sonicated and non-sonicated samples. The extract of the sonicated sample exhibited a more dark color (due to increased extraction of pigments). The release of pigments (chlorophyll and carotenoids) from algae has been used for evaluating the cell disruption under different sonication conditions [76].

Extraction took place by DMCHA at room temperature (18 ± 2 °C) using two different steps: mild stirring for 10 min in order to avoid extensive mixing and formation of emulsion, and extraction elaborating an extended time for 4 days without any stirring to reach equilibrium conditions without the formation of emulsion.

The organic solvent phase was then separated by centrifugation (6 min at 5000 rpm at room temperature) and 300 μL (15 times × 20 μL) of the DMCHA-extract solution were casted in filter paper sheets; replicates were received by deposition of half of the solution volume at two different spots. After drying, the paper sheet was placed in 5 L glass bottles containing 100 mL of solvent (hexane, ethyl acetate, DMCHA, and 1-pentanol). The containers were sealed and after 20–120 min the solvent impregnated the paper sheet to a predetermined height. Paper sheets were then air-dried at room temperature, and the upper fraction (the one that traveled up to the highest point along with the solvent) in the hexane chromatograph was examined by ATR-FTIR with 32 scans at 4 cm^−1^ resolution.

Besides these four solvents, an additional solvent mixture was used for paper chromatography, containing methanol/chloroform/water in volume ratios of 2/1/0.8, respectively. This mixture is the standard solvent used for total lipids determination in the Bligh and Dyer method [51].

### 4.3. Extraction of Fatty Acid Esters from Algae Liquor

First, 100 mL of algae liquor with a solids content of 1.5 ± 0.5 g/L was ultrasonicated for 30 min with power of 62.5 W/L and mixed with 100 mL of hexane. The two-phase mixture was subjected to mild magnetic stirring for 4 h at room temperature. A sample from the upper organic phase was examined by UV–Vis and ATR-FTIR spectroscopy with a resolution of 4 cm^−1^ and 32 scans. A dried algae sample was also examined by ATR-FTIR. A commercial edible sunflower oil was examined with ATR-FTIR and a 50% *v*/*v* solution of the sunflower in hexane was examined by UV–Vis. The extraction yield and the % solvent recovery were calculated by taking into account the solids content, the volume of the algal liquor, and mass of the extract measured after hexane distillation in a pre-weighted flask (in the same experiments the amount of recovered hexane was measured). The corresponding values represent the average ± standard deviation of four measurements.

Finally, in order to examine the composition of the extract using the Bligh–Dyer method [51] applied for the determination of total lipids, 1 g of wet paste (80% water) received after centrifugation (4900 rpm at room temperature) was mixed with 2 mL of chloroform and 4 mL of methanol and was magnetically stirred for 5 min. Then vacuum filtering was applied to remove the solids. The UV–Vis spectrum of the extract was recorded in the wavelength range 200–700 nm.

## 5. Conclusions

A simplified methodology based on Hansen Solubility Parameters was developed in this work aiming to perform solvent screening for selective extraction purposes. The method takes into account the affinity of the candidate solvent with the desired solutes, the lack of affinity of the solvent with non-desired solutes, the affinity with water, as well as the relative difference in these factors. The method was applied for the selective extraction of fatty acid esters from microalgae. More than 5000 substances were used for an initial assessment and classification of their suitability as potential selective lipid extraction solvents. Among the various solvents, hexane was predicted to be a good candidate. The prediction of hexane extraction capacity was further theoretically supported by ab initio DFT calculations of the Gibbs free energy of solvation and the corresponding hexane/water partition coefficient of various representative desired and non-desired solutes. The suitability of hexane was tested over four other solvents, three pure solvents (DMCHA, ethyl acetate, and 1-pentanol), and one solvent mixture (methanol/chloroform/water) commonly used for the determination of total lipid concentration. The superior performance of hexane was confirmed experimentally by paper chromatography, and it was found that hexane was the single solvent by which various lipid compounds are separated and eluted at different times/distances. Comparison of hexane results with the corresponding result from the solvents mixture revealed that the latter can result in an overestimation of the lipids content of microalgae due to the co-extraction of chlorophyll. Liquid–liquid extraction of microalgal liquor with hexane resulted in a low yield, but the extract was enriched in fatty acid esters.

## Figures and Tables

**Table 1:** HSP of desired and non-desired solutes [15,19].

Solutes	δ_d_, MPa^1/2^	δ_p_, MPa^1/2^	δ_hb_, MPa^1/2^
**desired solutes**			
**glycerides (fatty acid esters)**			
glyceryl Monostearate	16.1	4.5	9.8
glyceryl Monooleate	16.2	4.6	9.4
glyceryl Tributyrate	16.3	2.5	7.0
glyceryl Trioleate	16.0	3.8	3.2
**non-desired solutes**			
**sterols**			
β-sitosterol	17.2	1.8	3.4
**pigments**			
chlorophyll	20.2	15.6	18.2
lutein	17.8	1.5	5.1
**phospholipids**			
phosphatidylethanolamine	16.2	7.1	9.8
phosphatidylserine	17.6	12.2	18.7

**Table 2:** Overall score, Snyder’s polarity index, and dielectric constant for five candidate solvents for selective extraction of fatty acid esters from microalgae.

Solvent	Snyder’s Polarity Index [42]	Dielectric Constant [43]	Overall Score	Classification (Out of 5632 Solvents)
hexane	0	1.8865	12.653	188
DMCHA	triethyl amine 1.8	2.86 *	12.211	775
ethyl acetate	4.3	6.0814	11.910	1531
1-pentanol	isopentanol 3.6	15.13	11.143	4545
methanol/chloroform/water mixture in volume ratios of 2/1/0.8	chloroform 4.4	methanol 33	8.567	
	methanol 6.6	chloroform 4.8069		
	water 9	water 80.1		rejected

* this value was obtained from [44].

**Table 3:** Gibbs free energy of solvation of five representative solutes by hexane, 1-pentanol, and water.

Solute	ΔG of Solvation Hexane, kJ/Mol	ΔG of Solvation 1-Pentanol, kJ/Mol	ΔG of Solvation Water, kJ/Mol
glyceryl triolate	−169.8	−166.2	−23.2
β-sitosterol	−58.7	−63.9	−8.4
chlorophyll a	−158.0	−176.5	−88.9
phosphatidylethanolamine	−48.4	−91.9	−82.3
lutein	−102.9	−118.6	−46.9

**Table 4:** Hexane/water and 1-pentanol/water partition coefficients of five representative solutes.

Solute	log*p*_hexane/water_	log*p*_1-pentanol/water_
glyceryl triolate	25.70	25.06
β-sitosterol	8.80	9.72
chlorophyll a	12.10	15.35
phosphatidylethanolamine	−5.94	1.69
lutein	9.82	12.57

**Table 5:** Difference in solvation energies ΔΔG_solvation_ between the desired and non-desired solutes for various solvents calculated as ΔΔG_solvation_ = ΔG_solvation, desired solute_ − ΔG_solvation non-desired solute_.

Non-Desired Solute	ΔΔG_solvation_, Hexane, kJ/Mol	ΔΔG_solvation_, 1-Pentanol, kJ/Mol	ΔΔG_solvation_, Water, kJ/Mol
β-sitosterol	−111.2	−102.3	−14.8
chlorophyll a	−11.8	10.3	65.7
phosphatidylethanolamine	−121.4	−74.3	59.1
lutein	−66.9	−47.6	23.7

## Data Availability

The original contributions presented in this study are included in the article and Supplementary Materials. Further inquiries can be directed to the corresponding author.

## Supplementary Materials

The following supporting information can be downloaded at: https://www.mdpi.com/article/10.3390/molecules30224428/s1, Table S1: Comparison of COSMO-RS and HSP approaches for solvent screening; Table S2 (in excel file): Scores of various substances for their potential as solvents for selective extraction of glycerides from microalgae; Table S3: Overall score and Snyder’s polarity index for 70 solvents.
