# Supplementary material for: A Simplified Methodology for Solvent Screening in Selective Extraction of Lipids from Microalgae Based on Hansen Solubility Parameters
Source: Molecules. 2025 Nov 16;30(22):4428. doi: 10.3390/molecules30224428 (PMC12655419; doi:10.3390/molecules30224428)
Supplement: Supplementary file 1 [file molecules-30-04428-s001.zip › Supplementary Material S1 and S3.pdf]

Supplementary Material for

A Simplified Methodology for Solvent Screening in Selective Extraction of Lipids from Microalgae Based on Hansen Solubility Parameters

Costas Tsioptsias [1,*], Stefania Mitis [1], Alexandra Rentzela [1], Kalitsa Alvanou [1], Dimitra Kelesi [1], Christos Manolis [1], Anastasia Stergiou [1], Sotirios D. Kalamaras [2] and Petros Samaras [1]

[1] Department of Food Science and Technology, Alexandrian University Campus at Sindos, International Hellenic University, 57400 Thessaloniki, Greece; natasastergiou@hotmail.com (A.S.); samaras@ihu.gr (P.S.)
[2] Laboratory of Animal Production and Environmental Protection, Faculty of Veterinary Medicine, School of Health Sciences, Aristotle University of Thessaloniki, 54124 Thessaloniki, Greece; skalamaras@vet.auth.gr
* Correspondence: ktsiopts@ihu.gr

Table S1. Comparison of COSMO-RS and HSP approaches for solvent screening.

	**Thermodynamic models e.g. COSMO-RS, UNIFAC etc.**	**HSP**
Computational cost	high	low
Accuracy	high	low
Required expertise	high	low

Table S2. See attached excel file.

Table S3. Overall score and Snyder's polarity index for 70 solvents.

**Solvent**	**Snyder's Polarity index**	**Overall Score**
Squalane	-0.8	12.653
n-Decane	-0.3	12.643
Cyclohexane	0	12.573
n-Hexane	0	12.653
Carbon disulfide	1	9.756
Carbon tetrachloride	1.7	12.468
Dibutyl ether	1.7	11.849
Triethylamine	1.8	12.662
Diisopropyl ether	2.2	11.833
Toluene	2.3	12.385
p-Xylene	2.4	11.382
Chlorobenzene	2.7	12.185

Iodobenzene	2.7	10.730
Diphenyl ether	2.8	11.050
Ethoxybenzene	2.9	11.191
Diethyl ether	2.9	11.759
Benzene	3	12.349
Tricresyl phosphate	3.1	10.654
Ethyl bromide	3.1	11.225
n-Octanol	3.2	11.070
Fluorobenzene	3.3	11.231
Dibenzyl ether	3.3	10.944
Methylene chloride	3.4	11.094
Methoxybenzene	3.5	11.310
Isopentanol	3.6	10.369
Ethylene chloride	3.7	11.054
Bis(2-ethoxyethyl) ether	3.9	11.716
tert.-Butanol	3.9	11.085
n-Butanol	3.9	10.791
n-Propanol	3.9	8.154
Tetrahydrofuran	4.2	11.525
2.6-Lutidine	4.3	11.244
Ethyl acetate	4.3	11.910
Isopropanol	4.3	10.666
Chloroform	4.4	11.393
Acetophenone	4.4	10.817
Methyl ethyl ketone	4.5	11.049
Cyclohexanone	4.5	10.913
Nitrobenzene	4.5	11.370
Benzonitrile	4.6	10.305
Dioxane	4.8	11.288
2-Picoline	4.8	10.988
Tetramethylurea	5	9.335
Diethylene glycol	5	7.873
Triethylene glycol	5.1	7.897
Ethanol	5.2	7.900
Quinoline	5.2	10.711
Pyridine	5.3	10.639
Nitroethane	5.3	10.150
Acetone	5.4	11.685
Ethylene glycol	5.4	8.161
Benzyl alcohol	5.5	9.902
Methoxyethanol	5.7	10.686
Propylene carbonate	6	11.269
Oxydipropionitrile	6.2	9.881
Aniline	6.2	11.158
Methyl formamide	6.2	8.179

Acetic acid	6.2	11.033
Acetonitrile	6.2	11.021
N.N-Dimethylacetamide	6.3	11.174
Dimethyl formamide	6.4	7.596
Tetrahydrothiophene-1.1-dioxide	6.5	10.826
Dimethyl sulfoxide	6.5	8.712
N-Methyl-2-pyrrolidone	6.5	12.318
Hexamethyl phosphoric acid triamide	6.6	12.154
Methanol	6.6	8.517
Nitromethane	6.8	11.026
m-Cresol	7	10.876
Dodecafluoroheptanol	7.9	11.213
Water	9	8.094
